# Thin foil body-shield resuscitation barrier device to protect from blood: an experimental study

**DOI:** 10.1038/s41598-022-17915-4

**Published:** 2022-08-09

**Authors:** Martin Hermann, Markus Isser, Valentin Bilgeri, Andreas Klinger, Wolfgang Lederer

**Affiliations:** 1 Department of Anaesthesiology and Critical Care Medicine, Medical University of Innsbruck, Anichstr. 35, 6020 Innsbruck, Austria; 2 Medical Division, Austrian Mountain Rescue Service-Tyrol, Florianistr. 2, 6410 Telfs, Austria

**Keywords:** Translational research, Fluidics

## Abstract

In times of collective concern about pandemics, body-shield resuscitation barrier devices are more and more considered to protect against transmission of different pathogens between rescuers and patients. The objective of this experimental study was to investigate the characteristics of blood drops dispersed on the surface of four different foils suitable for blanketing patients during resuscitation. We analyzed run-off characteristics of blood stains depending on surface properties of polyvinyl chloride, polyethylene, polyethylene terephthalate and aluminum-coated polyethylene terephthalate. Confocal fluorescence microscopy revealed less cellular density and lack of fibrin networks in blood stains on the four foil surfaces than on paper towel. Delayed clotting went along with larger areas of contamination indicating a greater likelihood of coming into contact with potential germs but a smaller chance of contracting an infection. Space blankets as obligatory components of first aid kits are readily available for rescuers and serve as a mechanical barrier between rescuers and patients during resuscitation.

## Introduction

Unprotected rescuers are exposed to numerous pathogens during cardiopulmonary resuscitation (CPR), either through direct contact with the patient’s blood, body fluids or with droplets and aerosols^1^. Despite the ubiquitous risk of infection with human immunodeficiency virus (HIV), hepatitis B virus (HBV) and hepatitis C virus (HCV) through blood, current attention is focused mainly on transmission of viruses by aerosols and droplets during CPR^1^.

During the corona virus disease (COVID-19) pandemic, the fear of contracting severe acute respiratory syndrome-associated corona virus (SARS-CoV-2) has become the prime reason for lay rescuers to not perform CPR and for bad resuscitation outcome^2,3^. Consequently, the European Resuscitation Council omitted ventilation attempts by lay rescuers and recommended compression-only CPR and public-access defibrillation (PAD) during the COVID-19 pandemic^4^. Although the potential for contracting HIV, HBV or HCV via saliva through mouth-to-mouth ventilation is extremely low, viruses can be contracted by direct contact with blood and wounds^5^. Since even the smallest volumes of blood in infected persons may contain infectious viruses, the quantity of exposed blood plays a role in relation to the likelihood of rescuers being infected through direct contact^6^.

Basic infection-control measures such as plastic shields as a barrier between patient and rescuer are expected to diminish contaminations from vomit, blood and body secretions^7^. Presumably, the strong hydrophobicity of plastic foils may have additional effects on infectious secretions beside the barrier function^8^. Foils may even reduce revulsion, thus increasing the readiness of lay rescuers to perform CPR^9^.

The object of this experimental study was to investigate the dispersion of fresh blood stains on the surface of four different foils suitable for blanketing patients during CPR.

## Results

Foil fragments of either PVC, PE, PET, and Al-PET were investigated according to clotting and run-off speed from 30 to 100 µl drops of untreated blood. Six blood drops freshly drawn from a co-author were investigated per foil and volume (total 48 runs).

### CFM investigation

**Figure 1:**
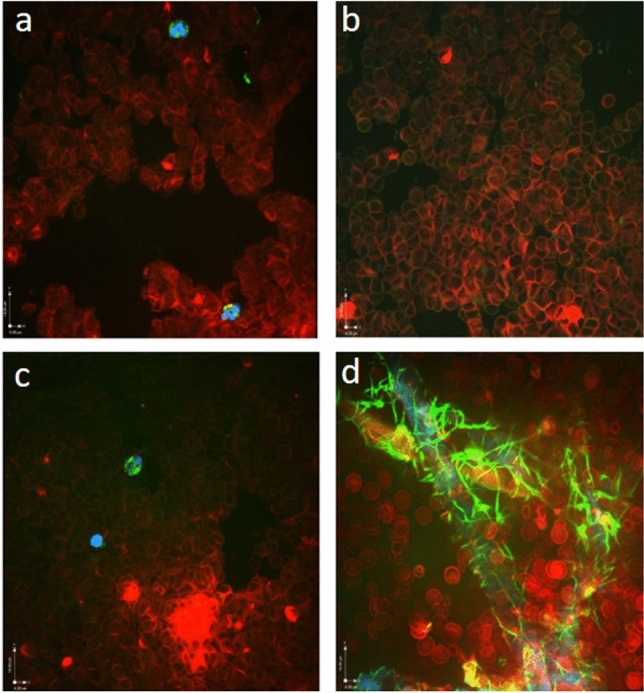
Real time live confocal imaging of human blood (objective 40 × water immersion), displaying aggregated and dried erythrocytes stained with wheat germ agglutinin-Alexa Fluor 555 and fluorescein isothiocyanate-linked Fibrin-Binding Peptide (FITC-FFBP). Cell nuclei, morphology and fibrin were visualized via HOECHST staining (blue). (**a**) Aluminum-coated polyethylene terephthalate: No fibrin network was detected using FFBP solution. Nuclei of two lymphocytes are visible at the top and bottom of the image. Both lymphocytes contain internalized FFBP positive vesicles. (**b**) Polyvinyl chloride: No fibrin network was detected using FFBP solution. (**c**) Polyethylene: at the center of the image the nucleus of a lymphocyte visualized via HOECHST staining (blue), containing internalized FFBP-positive vesicles. No fibrin network was detected using FFBP solution. (**d**) Paper towel: numerous fibrin networks (green) and erythrocytes (red). Notably, most of the fibrin fibers were in close contact with the paper towel fibers.

Real time live confocal imaging revealed fibrin formation only in the paper towel controls but not in the foils (Fig. 1).

### Run-off area and run-off speed

**Figure 2:**
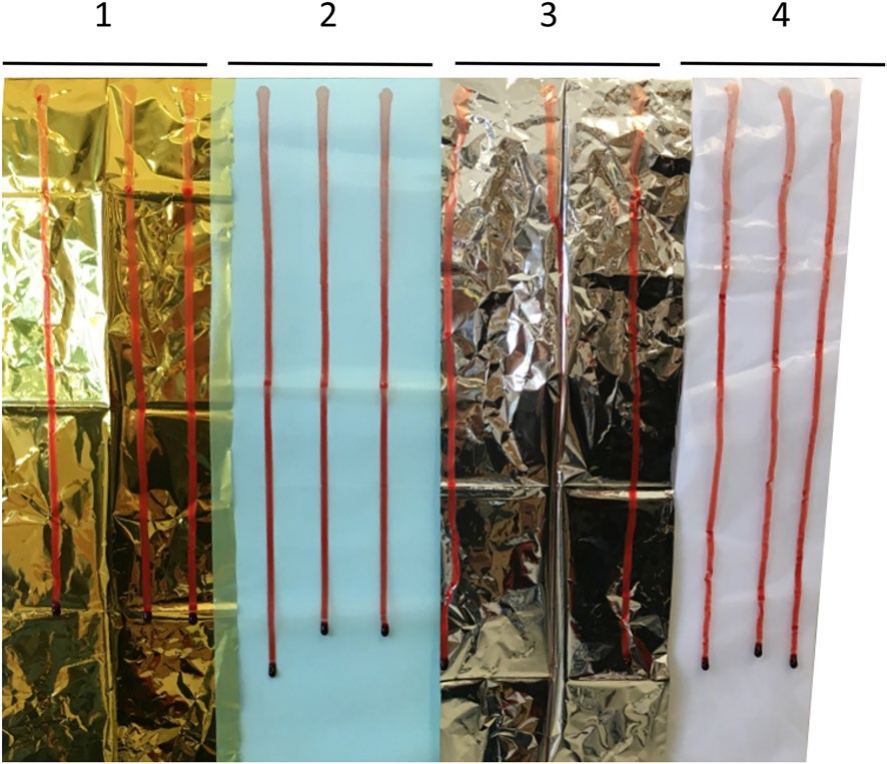
Blood stain: 10 min run-off pattern of twelve 100 µl drops on vertically arranged (**1**) polyethylene terephthalate (PET), (**2**) polyethylene (PE), (**3**) aluminum-coated polyethylene terephthalate (Al-PET) and (**4**) polyvinyl chloride (PVC) stripes.

The diameter of 30 µl and 100 µl drops and lengths of run-off drainage marks at 2, 7, 10, 20 and 30 min were measured (Fig. 2). Overall, lengths of drainage marks and run-off speed were higher with 100 µl drops than with 30 µl drops. Drainage marks in 30 µl drops ranged between 8 and 75 mm with few alterations after 10 min. Similarly, in 100 µl drops drainage marks exceeded 300 mm on all tested foils within the first 10 min. After an initial continuous drainage of 100 µl drops the further run-off occurred gradually in intervals showing few alterations between 20 and 30 min. Run-off speed within 2 min ranged between 2.7 and 2.8 mm/s. Clotting was significantly delayed and the ratio between end drop area and run-off area as surrogate of presumed infectivity was diminished to approx. 1% on all tested foils (Table 1).

**Table 1:** Mean run-off diameter after intervals of 2, 7, 10, 20 and 30 min, mean drop area, mean run-off area and mean run-off speed for 100 µl drops in vertically arranged stripes of polyvinyl chloride (PVC), polyethylene (PE), polyethylene terephthalate (PET) and aluminum-coated polyethylene terephthalate (Al-PET).

	PVC	PE	PET	Al-PET
	m ± SD	m ± SD	m ± SD	m ± SD
**Blood stain length**				
2 min run-off length (mm)	296 ± 20.4	235 ± 27.2	253 ± 19.4	266 ± 8.6
7 min run-off length (mm)	365 ± 32.2	318 ± 24.3	347 ± 16.3	315 ± 38.6
10 min run-off length (mm)	378 ± 35.7	328 ± 21.8	378 ± 40.1	324 ± 65.0
20 min run-off length (mm)	383 ± 36.3	335 ± 30.3	394 ± 48.0	330 ± 72.9
30 min run-off length (mm)	385 ± 37.0	335 ± 30.3	396 ± 48.5	344 ± 71.0
**Blood stain width**				
30 min initial drop width (mm)	8.2 ± 1.2	7.5 ± 0.8	9.2 ± 0.8	9.2 ± 0.6
30 min run-off mid-width (mm)	4.1 ± 0.6	3.8 ± 0.7	4.3 ± 0.4	4.3 ± 0.4
30 min end drop width (mm)	3.7 ± 0.5	3.6 ± 0.5	4.2 ± 0.4	4.7 ± 0.8
**Run-off speed and blood stain area**				
2 min run-off speed (mm/s)	2.5 ± 0.2	2.0 ± 0.2	2.1 ± 0.2	2.2 ± 0.1
30 min run-off area (mm^2^)	1,586 ± 347	1,321 ± 144	1,703 ± 339	1,481 ± 355
Initial drop area (mm^2^)	53.3 ± 15.5	44.6 ± 10.4	66.4 ± 10.8	66.4 ± 10.8
End drop area (mm^2^)	10.7 ± 2.8	11.2 ± 2.6	14.0 ± 3.2	14.4 ± 9.4
**Contamination**				
Initial drop area/run-off area (%)	2.6 ± 1.1	2.9 ± 0.3	4.1 ± 1.3	4.8 ± 1.7
**Presumed infectivity**				
End drop area/initial drop area (%)	22.6 ± 10.3	29.1 ± 7.2	22.3 ± 5.8	22.7 ± 16.1
End drop area/run-off area (%)	0.7 ± 0.1	0.8 ± 0.1	0.8 ± 0.2	1.1 ± 0.7

## Discussion

To our knowledge this is the first experimental study to compare different materials suitable for barrier resuscitation regarding their potential to diminish exposure to blood. As the quantity of exposed blood differs within the run-off figure of a blood stain, the likelihood of infection depends on direct contact with either blood drops or large run-off areas. Even before the pandemic began, fear of infection was a significant reason for lay rescuers to not perform CPR^10^. The use of personal protective equipment (PPE) to minimize the risk of infection was of utmost importance during the pandemic^11^. Unfortunately, accessibility to PPE is limited to professional rescue teams. Thin foils were established in first aid kits or at public defibrillators for the purpose of protecting lay rescuers performing resuscitation.

Our experimental results revealed that clotting was delayed on all tested materials as shown by the lack of fibrin networks in comparison to paper towels. Initial blood drops differed in area and diameter according to the different hydrophobicity of foils. We consider hydrophobicity and delayed clotting to be related to surface properties of the test materials^8,12^. Since the prosthetic blood interaction depends on the nature of the surface, the wide choice of additives, ingredients, plasticizers, fillers, and stabilisers used with PVC affects blood coagulation^12^. Lowkis and Szymomowicz reported that fresh, human citrate blood on PVC had a markedly prolonged thromboelastographic coagulation time and time of clot formation to standard elasticity. The authors discussed the potential effect of electric charge on the adhesion of human blood platelets^13^. Corresponding with our findings, coating with aluminum oxide (Al_2_O_3_) was reported to further increase anticoagulation effects^14^.

Our experimental investigation showed that, on the one hand, delayed clotting on different foil surfaces goes along with larger areas of contamination, which increases the chance of coming into contact with potential germs. On the other hand, the lesser content of germs per area decreases the likelihood of contracting an infection. The foil as mechanical barrier between rescuer and patient provides an additional protective effect. Length of run-off marks was more pronounced on both sides of the space blanket, indicating that smooth surface texture influenced dispersion of blood more than did composition of foils.

There should be a diminished risk of contamination from blood when the foil is used as barrier between rescuer and patient. It is likely that the faster drainage of blood on the space blanket leads less germ load to be expected on exposed sites, thus reducing the risk of contamination for rescuers. In addition, survival of human coronaviruses was reported to be significantly shorter on aluminum surfaces than on PVC surfaces^15^.

Blanketing the patient with a foil can protect against contamination by blood, vomit and secretions. Whereas PVC and PE foils may not be readily available to lay rescuers, space blankets as basic components of first aid kits are. Ideally, space blankets used for thin film barrier resuscitation would need a transparent center to facilitate finding the correct pressure point for resuscitation. For professional rescuers foils with a predesigned central perforation could help ensure air-tight installation of mask or tube with filter when ventilation is attempted. Eventually, invasive procedures with direct blood contact e.g. venipuncture or chest drainage are allocated to professional staff additionally equipped with PPE^11^. Presumably, covering the patient with a space blanket can reduce the risk of contamination and may prompt an even greater willingness of lay rescuers to start resuscitation. This, however, deserves further clinical investigation.

There are several limitations to be accounted. We performed preliminary investigations comparing blood on glass slides and on test slides to detect potential interference from the interventions. Blood was not evenly spread in run-off figures. This was particularly obvious on space blankets with their package-dependent longitudinal and transverse folds. This made dispersion of blood more unpredictable. We are aware that infectivity depends on exceeding the lower threshold of germ load and that it does not necessarily increase in a linear manner with increasing germ load. For the sake of simplification, let us state that we postulated germs to be distributed equally in blood fluid and germ load to linearly correlate with the estimated area of contamination.

In conclusion, thin film barrier resuscitation could protect against pathogens transmitted by skin contact, aerosol or droplet infection. On the one hand, delayed clotting might increase the risk of contamination from direct contact. On the other hand, the lesser concentration of germs in the run-off area that goes along with increased fluid surface and decreased volume might indicate less risk of infection. Applications and mode of handling by lay rescuers need to be investigated to determine the optimal choice of barrier material. It would be worthwhile to see whether a barrier between patient and rescuer might even increase the willingness of lay rescuers to start CPR.

## Materials and methods

### Study design and ethics considerations

Run-off characteristics of blood drops on various foil surfaces were macroscopically observed. In addition, confocal fluorescence microscopy (CFM) was used to microscopically analyze cellular density and coagulation activity of blood stains on different foil surfaces. Investigations were conducted at the Anesthesiology Research Laboratory, Medical University of Innsbruck, Austria. Study design and manuscript presentation followed the SQUIRE 2.0 Revised Standards for Quality Improvement Reporting Excellence to improve the quality, safety, and value of healthcare^16^. As the experimental design focused on physical properties of plastic foils and not on patients and users the institutional guidelines of the OE Clinical Trial Center (OE CTC), Medical University of Innsbruck, did not advocate the need for ethics approval.

### Context

Surface properties of four different materials potentially applicable for blanketing patients during CPR were investigated.

### Intervention

Specifics of the team involved anaesthesiology and critical care (Lederer W, Hermann M; Bilgeri V) and mountain rescue (Isser M, Klinger A).

### Test materials

We investigated four easily accessible foils, waterproof, low-weight, low-bulk with varying transparency and surface structure.

1. Plasticised polyvinyl chloride (PVC) foil (d50 universalpresenning; JUFOL GmbH, Krumbacher Str. 9, D-86154, Augsburg, Germany), smooth surface, with an area of 4000 × 5000 cm and a thickness of 50 μm.
2. Polyethylene (PE) foil (Henry Schein Inc., 135 Duryea Rd., Melville, NY 11747, USA), rough and structured surface, with an area of 120 × 120 cm.
3. Polyethylene terephthalate (PET) surface and
4. 1% aluminum-coated PET blanket (Al-PET) surface of a space blanket (First Aid Blanket; M06299; Franz Kalff GmbH, D-53881 Euskirchen, Germany), smooth surface, with an area of 160 × 210 cm, 0.012 mm in thickness, approx. 50 g in weight.
5. Bleached pulp, absorbent, rather bulky with rough and structured surface (Paper towel; Zentrale Handelsgesellschaft GmbH, D-77656 Offenburg, Germany) served as control.

### Measuring device

CFM (Zeiss Observer Z1, Zeiss, Oberkochen, Germany) in arrangement with a spinning disc confocal system (UltraVIEW VoX, Perkin Elmer, Waltham, MA, USA) was used to perform high-resolution imaging from blood contamination on four surfaces of selected barrier materials. The technique combined a focused laser for excitation with a pinhole for detection for three-dimensional imaging. It provided high-sensitivity, low-phototoxicity and low noise hybrid detection^17^.

### Experimental setup

In a self-constructed physical model we attached four longitudinal 200 × 6000 mm stripes of PVC, PE, PET, and Al-PET to a frame. Three freshly drawn blood drops per stripe were applied on the upper edge using an Eppendorf pipette. Then the frame was brought to a vertical position and the run-off characteristics of the blood stains were observed at 2-, 5-, 7-, 10-, 20- and 30-min intervals. The investigation was performed for 30 µl drops and for 100 µl drops and repeated once.

In a different experimental setting we used CFM to microscopically visualize changes in stained blood drops after run-off on the surface of the four tested materials. For preparation of the blood live stain mixture we added 10 μg/ml fluorescently labeled wheat germ agglutinin Alexa Fluor 555 (Molecular Probes, Waltham MA, USA), Hoechst (Molecular Probes) and 4.5 μg/ml fluorescent fibrin-binding peptide solution (FFBP; Genic Bio, Shanghai, China) to 1 ml of fresh whole blood^18^. Similar to the initial experiment the run-off areas from 30 µl drops and 100 µl drops were investigated. At the end of the test run 24 × 50 mm specimens of the four shields were taken, mounted on microscope slides (Menzel-Gläser, Vienna, Austria) and microscopically examined with real time live confocal imaging at three wavelengths (405/488/561 nm). Blood drops trickled on paper towel served as control. Density and dispersion of fibrin networks and red blood cells were compared. Observations were repeated three times per foil. Investigations were made at room conditions of 31.4% relative humidity and 23.3 °C temperature.

### Study of the interventions

The run-off characteristics of 30 µl and 100 µl untreated blood drops on PVC, PE, PET, and Al-PET surfaces were analyzed by measuring lengths and width of drainage marks (in mm) 2, 7, 10, 20 and 30 min after the frame was brought to a vertical position. The initial run-off speed was calculated (in mm/s) during the first two minutes of observation. After 30 min we measured run-off mid-width and length as well as initial and end drop diameters of each blood stain. The corresponding areas for the entire run-off (mid-width × length), and the initial drop and the end drop ((diameter/2)^2^ × π) were calculated (in mm^2^).

Assuming equal distribution of germs in blood fluid during infection, the germ load per area is higher in accumulated blood drops than in dispersed blood after run-off. In order to estimate the relative germ load in the run-off area (A_run_) we calculated the reduced area of the end drop related to the area of the initial drop. The ratio of the area of the end drop (A_end_) to the area of the initial drop (A_init_) indicates the reduced area of contact (AC) with high germ load (1). AC values were multiplied by 100 to express results as a percentage.

1 $$ {\text{AC }} = {\text{A}}_{{{\text{end}}}} /{\text{A}}_{{{\text{init}}}} . $$

The ratio of initial drop area to run-off area was calculated to indicate the area gain (AG) of increased contamination (2). AG was expressed as a percentage.

2 $$ {\text{AG }} = {\text{A}}_{{{\text{init}}}} /{\text{A}}_{{{\text{run}}}} . $$

Area equivalent (AE) ratio was calculated by ratio multiplication as the product of AC multiplied by AG. Based on the assumption that germs are equally distributed in blood fluid, the sum germ load on run-off surfaces and in the end drop equals germ load in the initial drop. Thus, the AE ratio indicates the reduced germ load per area as surrogate of presumed infectivity (3). The hypothetical germ load after run-off was expressed as a percentage.

3 $$ {\text{AE }} = \, \left( {{\text{A}}_{{{\text{end}}}} /{\text{A}}_{{{\text{init}}}} } \right) \, \times \, \left( {{\text{A}}_{{{\text{init}}}} /{\text{A}}_{{{\text{run}}}} } \right) \, = {\text{A}}_{{{\text{end}}}} /{\text{A}}_{{{\text{run}}}} . $$

Via CFM we visualized fibrin and cellular structures in real time using a 10 × objective for the overview and a 40 × water immersion objective with a numerical aperture of 1.4 for a more detailed view. On PET and Al-PET we used bright field microscopy (objective 40 × water immersion) to visualize erythrocytes. We used HOECHST staining to visualize nuclei of leukocytes and lymphocytes. Extrinsic pathway of coagulation was induced by adding STAR-TEM and EXTEM reagents from rotational thromboelastometry (ROTEM).

### Measures

Rationale for choosing the four materials was their availability in the out-of-hospital setting. In accordance with sample size estimation six test runs were performed per foil and per selected volume of 30 µl and 100 µl blood. With respect to material and volume of the initial blood drop we observed little variation in the data. Prior to the investigation we performed preliminary investigations comparing blood on glass slides and on test slides to detect potential interference from the interventions. Elements that contributed to, influenced and altered efficiency were humidity and room temperature.

### Statistical analysis

Descriptive statistics were applied using the statistical package for the social sciences (IBM SPSS Statistics Standard Version 27, Armonk, NY, USA) to determine mean and standard deviation. The estimated sample size was minimum six runs per group to achieve a power of 80% (power = 0.8) and a level of significance of 5% (alpha = 0.05) with one-way analysis of variance for means between 250 and 30 and standard deviation of 25 using an online sample size calculator (https://homepage.univie.ac.at/robin.ristl/samplesize.php?test=wilcoxon).

## Data Availability

All data generated or analysed during this study are included in this published article.
